# Supplementary material for: The early-acting glycosome biogenic protein Pex3 is essential for trypanosome viability
Source: Life Sci Alliance. 2019 Jul 24;2(4):e201900421. doi: 10.26508/lsa.201900421 (PMC6658674; doi:10.26508/lsa.201900421)
Supplement: Supplementary file 1 [file LSA-2019-00421_TableS1.docx]

Table S1. Amino acid identity matrix between Pex3 proteins

|  | HsPex3 | AtPex3 | ScPex3 | PfPex3 | NgPex3 | LmPex3 | TbPex3 |
| --- | --- | --- | --- | --- | --- | --- | --- |
| HsPex3 | **100.00** | 27.57 | 18.31 | 24.78 | 22.32 | 12.79 | 11.94 |
| AtPex3 | 27.57 | **100.00** | 19.02 | 22.39 | 20.54 | 10.68 | 11.30 |
| ScPex3 | 18.31 | 19.02 | **100.00** | 17.66 | 13.25 | 12.76 | 9.46 |
| PfPex3 | 24.78 | 22.39 | 17.66 | **100.00** | 23.17 | 10.51 | 11.01 |
| NgPex3 | 22.32 | 20.54 | 13.25 | 23.17 | **100.00** | 14.32 | 10.61 |
| LmPex3 | 12.79 | 10.68 | 12.76 | 10.51 | 14.32 | **100.00** | 28.17 |
| TbPex3 | 11.94 | 11.30 | 9.46 | 11.01 | 10.61 | 28.17 | **100.00** |

Percent identity of query sequence with itself is shown in bold.
