# Supplementary material for: The early-acting glycosome biogenic protein Pex3 is essential for trypanosome viability
Source: Life Sci Alliance. 2019 Jul 24;2(4):e201900421. doi: 10.26508/lsa.201900421 (PMC6658674; doi:10.26508/lsa.201900421)
Supplement: Supplementary file 2 [file LSA-2019-00421_TableS2.docx]

Table S2. Orthologues and paralogues of Pex3 within the Kinetoplastida*

| **Gene** | **Organism** | **Annotation** | **Is Syntenic** |
| --- | --- | --- | --- |
| BCY84_11776 | *Trypanosoma cruzi* Dm28c 2017 | hypothetical protein | yes |
| Baya_134_0190 | *Blechomonas ayalai* B08-376 | hypothetical protein, conserved | yes |
| C3747_315g13 | *Trypanosoma cruzi* TCC | hypothetical protein, conserved | yes |
| C4B63_40g48 | *Trypanosoma cruzi* Dm28c 2018 | hypothetical protein, conserved | yes |
| CFAC1_280044800 | *Crithidia fasciculata* strain Cf-Cl | hypothetical protein, conserved | yes |
| DQ04_08871000 | *Trypanosoma grayi* ANR4 | hypothetical protein | yes |
| EMOLV88_360046900 | *Endotrypanum monterogeii* strain LV88 | hypothetical protein, conserved | yes |
| LAEL147_000853300 | *Leishmania aethiopica* L147 | hypothetical protein, conserved | yes |
| LAMA_000347200 | *Leishmania amazonensis* MHOM/BR/71973/M2269 | hypothetical protein, conserved | yes |
| LARLEM1108_360050000 | *Leishmania arabica* strain LEM1108 | hypothetical protein, conserved | yes |
| LBRM2903_350053000 | *Leishmania braziliensis* MHOM/BR/75/M2903 | hypothetical protein, conserved | yes |
| LENLEM3045_360049800 | *Leishmania enriettii* strain LEM3045 | hypothetical protein, conserved | yes |
| LGELEM452_360049900 | *Leishmania gerbilli* strain LEM452 | hypothetical protein, conserved | yes |
| LINF_360049200 | *Leishmania infantum* JPCM5 | hypothetical protein, conserved | yes |
| LMARLEM2494_360049600 | *Leishmania sp.* MAR LEM2494 | hypothetical protein, conserved | yes |
| LMJLV39_360051100 | *Leishmania major* strain LV39c5 | hypothetical protein, conserved | yes |
| LMJSD75_360051000 | *Leishmania major* strain SD 75.1 | hypothetical protein, conserved | yes |
| LPAL13_350049400 | *Leishmania panamensis* MHOM/COL/81/L13 | hypothetical protein, conserved | yes |
| LPMP_354120 | *Leishmania panamensis* strain MHOM/PA/94/PSC-1 | hypothetical protein | yes |
| LTRL590_360050900 | *Leishmania tropica* L590 | hypothetical protein, conserved | yes |
| LTULEM423_360050300 | *Leishmania turanica* strain LEM423 | hypothetical protein, conserved | yes |
| LbrM.35.4250 | *Leishmania braziliensis* MHOM/BR/75/M2904 | hypothetical protein, conserved | yes |
| [LdBPK.36.2.004210](https://tritrypdb.org/tritrypdb/app/record/gene/LdBPK.36.2.004210) | *Leishmania donovani* strain LV9 | hypothetical protein, conserved | yes |
| [LdBPK_364210.1](https://tritrypdb.org/tritrypdb/app/record/gene/LdBPK_364210.1) | *Leishmania donovani* BPK282A1 | hypothetical protein, conserved | yes |
| LdCL_360049200 | *Leishmania donovani* CL-SL | hypothetical protein | yes |
| LmjF.36.4010 | *Leishmania major* strain Friedlin | hypothetical protein, conserved | yes |
| LmxM.36.4010 | *Leishmania mexicana* MHOM/GT/2001/U1103 | hypothetical protein, conserved | yes |
| LpyrH10_06_1720 | *Leptomonas pyrrhocoris* H10 | hypothetical protein, conserved | yes |
| Lsey_0094_0090 | *Leptomonas seymouri* ATCC 30220 | hypothetical protein, conserved | yes |
| LtaP36.4130 | *Leishmania tarentolae* Parrot-TarII | hypothetical protein, conserved | yes |
| PCON_0042250 | *Paratrypanosoma confusum* CUL13 | hypothetical protein | yes |
| TCDM_02250 | *Trypanosoma cruzi* Dm28c 2014 | hypothetical protein | yes |
| TCSYLVIO_008194 | *Trypanosoma cruzi* Sylvio X10/1-2012 | hypothetical protein | yes |
| TM35_000063870 | *Trypanosoma theileri* isolate Edinburgh | hypothetical protein | yes |
| TRSC58_05613 | *Trypanosoma rangeli* SC58 | hypothetical protein | yes |
| Tb427_110111200 | *Trypanosoma brucei* Lister strain 427 2018 | hypothetical protein, conserved | yes |
| Tb427tmp.01.2020 | *Trypanosoma brucei* Lister strain 427 | hypothetical protein, conserved | yes |
| Tb927.11.10260 | *Trypanosoma brucei brucei* TREU927 | hypothetical protein, conserved | yes |
| TcCLB.506275.40 | *Trypanosoma cruzi* CL Brener Non-Esmeraldo-like | hypothetical protein, conserved | yes |
| TcCLB.510719.280 | *Trypanosoma cruzi CL* Brener Esmeraldo-like | hypothetical protein, conserved | yes |
| TcIL3000.11.10780 | *Trypanosoma congolense* IL3000 | hypothetical protein, conserved | yes |
| Tc_MARK_7135 | *Trypanosoma cruzi marinkellei* strain B7 | hypothetical protein, conserved | yes |
| TevSTIB805.11_01.10570 | *Trypanosoma evansi* strain STIB 805 | hypothetical protein, conserved | yes |
| TvY486_1111120 | *Trypanosoma vivax* Y486 | hypothetical protein, conserved | yes |
| C3747_76g237 | *Trypanosoma cruzi* TCC | hypothetical protein, conserved | no |
| TcIL3000_0_42040 | *Trypanosoma congolense* IL3000 | hypothetical protein, conserved | no |

*Sourced from the Eukaryotic Pathogen Database (EuPathDB) (https://eupathdb.org/eupathdb/)
